# Association Between Parkinson's Disease and Atrial Fibrillation: A Population-Based Study

**DOI:** 10.3389/fneur.2019.00022

**Published:** 2019-02-05

**Authors:** Chien-Tai Hong, Lung Chan, Dean Wu, Wan-Ting Chen, Li-Nien Chien

**Affiliations:** ^1^Department of Neurology, Shuang Ho Hospital, Taipei Medical University, Taipei, Taiwan; ^2^Department of Neurology, School of Medicine, College of Medicine, Taipei Medical University, Taipei, Taiwan; ^3^Health and Clinical Data Research Center, College of Public Health, Taipei Medical University, Taipei, Taiwan; ^4^School of Health Care Administration, College of Management, Taipei Medical University, Taipei, Taiwan

**Keywords:** Parkinson's disease, atrial fibrillation, population-based study, autonomic nerve system, biomarker

## Abstract

**Introduction:** Autonomic nervous system (ANS) dysfunction contributes to several non-motor symptoms of Parkinson's disease (PD). In addition, ANS plays a role in the genesis and maintenance of atrial fibrillation (AF). This study investigated the temporal association between PD and AF.

**Methods:** Data were obtained from the National Health Insurance Research Database of Taiwan. In total, 15,375 patients with newly diagnosed PD were matched with four controls each based on the propensity score. This study was bidirectional. A case-control study for the odds ratio (OR) of AF before PD and within 2 years of PD diagnosis was evaluated through conditional logistic regression. Furthermore, a cohort study on the subdistribution hazard ratio (SHR) for new-onset AF 2 years after PD diagnosis was evaluated using competing risk analysis.

**Results:** In the case-control study, PD was found to be significantly comorbid with AF (adjusted OR: 1.15, 95% confidence interval [CI]: 1.04–1.28). Subgroup analysis demonstrated that this association consistently presented in the absence of confounding factors of AF. In the cohort study, people with PD were found to have a lower risk of AF (adjusted SHR: 0.92, 95% CI: 0.86–0.98). However, a consistent association was not observed between the confounding factors of AF and PD during the subgroup analysis.

**Conclusions:** This study demonstrated that the premotor and early stages of PD were comorbid with AF, whereas the risk of AF was lower in the later stages. Thus, AF might be a premotor predictive biomarker and comorbidity of early PD.

## Introduction

Tremor, bradykinesia, rigidity, and postural instability are the cardinal motor symptoms of Parkinson's disease (PD). However, numerous non-motor symptoms (NMSs), such as depression, dementia, rapid eye movement sleep behavior disorder (RBD), and anosmia, are also comorbid with PD (1). The biological basis of NMSs are distinct from those of conventional motor symptoms. Motor symptoms result from the degeneration of dopaminergic neurons in the midbrain substantia nigra, whereas NMSs result from dysfunction of the serotonergic, cholinergic, and catecholaminergic systems (2). On the basis of these clinical and pathological findings, PD is presently recognized as a disease involving multiple systems and neurotransmitters (3).

Degeneration of the autonomic nervous system (ANS) contributes to certain NMSs of PD, the best-known of which is constipation. More than 60% of people with PD develop constipation because of poor intestinal peristalsis caused by a dysfunctional vagus nerve. Moreover, in most cases, constipation heralds the onset of motor symptoms (4). This sequential association has been supported by a postmortem study. Aggregated α-synuclein, the pathological marker of PD, was first identified in the mesenteric plexus in a preclinical study with PD models. The medullary vagal nucleus is the first area in the central nervous system (CNS) to accumulate α-synuclein, which echoes hypothesis regarding caudal–rostral spreading of the Lewy body (5). Today, constipation and other ANS-related NMSs are recognized as possible predictive biomarkers of PD (6).

Cardiac rhythm is regulated by ANS as well. Sympathetic and parasympathetic innervations originate from the paravertebral ganglia and vagal nerves, respectively. Atrial fibrillation (AF) is the most common sustained cardiac arrhythmia, and it is strongly associated with morbidity, mortality, and poor quality of life. AF stems from several etiologies, and rather than ischemic heart disease, heart failure, and hyperthyroidism, ANS plays a crucial role in AF, particularly for patients with no structural heart disease (7, 8).

Considering the role of the vagal nerve–related ANS system in PD and AF, PD may be comorbid with AF. Moreover, similar to other autonomic NMSs, AF may be a biomarker for the onset of PD motor symptoms. This study employed the National Health Insurance Research Database (NHIRD) of Taiwan to investigate whether AF is associated with newly diagnosed PD and evaluated the temporal relationship between both conditions.

## Methods

### Institutional Review Board

This study was approved by the Joint Institutional Review Board of Taipei Medical University (TMU-JIRB No. 201701058).

### Data Source and Study Design

This study was conducted using the NHIRD data files maintained by the Health and Welfare Data Science Center (HWDC). The NHIRD is a claims-based database managed by the National Health Insurance Administration of Taiwan; Taiwan's NHI provides coverage for 99% of its residents. The NHIRD files include inpatient, outpatient, and pharmaceutical claims and disease diagnoses coded according to the International Classification of Diseases, Ninth Revision, Clinical Modification (ICD-9-CM). In addition, the enrollment files of beneficiaries and providers were also included. The data in this study were from 2000 to 2015. Additionally, we linked the collected data with the national death registry to obtain death records. The two data sets can be linked according to the regulations of the HWDC. Both case-control (diagnosed AF before and within a 2-year interval of the first PD diagnosis) and cohort (newly diagnosed AF 2 years after first PD diagnosis) studies were applied to examine the temporal relationship between PD and AF.

### Participants

Newly diagnosed people with PD were defined as those who had at least two diagnostic claims (ICD-9-CM: 332.0) and prescription claims for dopaminergic agents between 2004 and 2011. The index date of PD was defined as the date of first PD diagnosis, hereafter referred to as the index PD. People who were aged < 45 years, had a history of stroke, or had received any antipsychotic drug before the index PD, were excluded to avoid the possibility of misclassification of secondary Parkinsonism. In addition, several predisposing factors may trigger AF directly (9). Therefore, we also excluded people with a history of rheumatic heart disease (ICD-9-CM: 390–398), other structural heart disease (ICD-9-CM: 420–425), thyroid disease (ICD-9-CM: 240–246), diseases of the adrenal gland (ICD-9-CM: 255), alcoholism (ICD-9-CM: 303), and AF onset within 6-month intervals of severe acute infection (ICD-9-CM: 995.9) and cardiothoracic/abdominal surgery (ICD-9 procedure codes of 30–39 and 42–75). The same exclusion criteria were used for the control participants.

### Propensity Score Model

Matching aims to reduce potential selection bias in the observational studies. Propensity score matching (PSM) are widely utilized to diminish the confounding factors that inevitably occurs in studies investigating the effect of the exposures on the outcome. In PSM, matched are formed by virtue of sharing similar propensity score values. The weighted-value reveals the risk of the participant for the outcome of interest according to their underlying characteristics predispose them for that outcome irrespective of the exposure of interest (10).

In this study, the propensity score was measured on the basis of hypertension (HTN, ICD-9-CM: 401–405), diabetes mellitus (DM, ICD-9-CM: 250), hyperlipidemia (ICD-9-CM: 272), chronic heart failure (CHF, ICD-9-CM: 428), coronary artery disease (CAD, ICD-9-CM: 410–414), chronic lung disease (ICD-9-CM:415–417, 490–496, and 500–508), renal disease (ICD-9-CM: 580–589), and inflammatory dx (ICD-9-CM: 710, 714). The selection of these factors was based on the association with AF genesis (9). The effect of each factors on the AF was assessed by logistic regression (Supplementary Table 1). Controls without PD were assigned an index date of pseudo-PD diagnosis corresponding to the index date of PD diagnosis of their matched patients. Each person with PD was matched with four controls without PD based on age, sex, pseudo diagnostic year and the propensity score using a caliper of width equal to 0.2; consequently, the two cohorts had similar baseline characteristics but differed in PD diagnosis.

### Study Outcome

**Figure 1:**
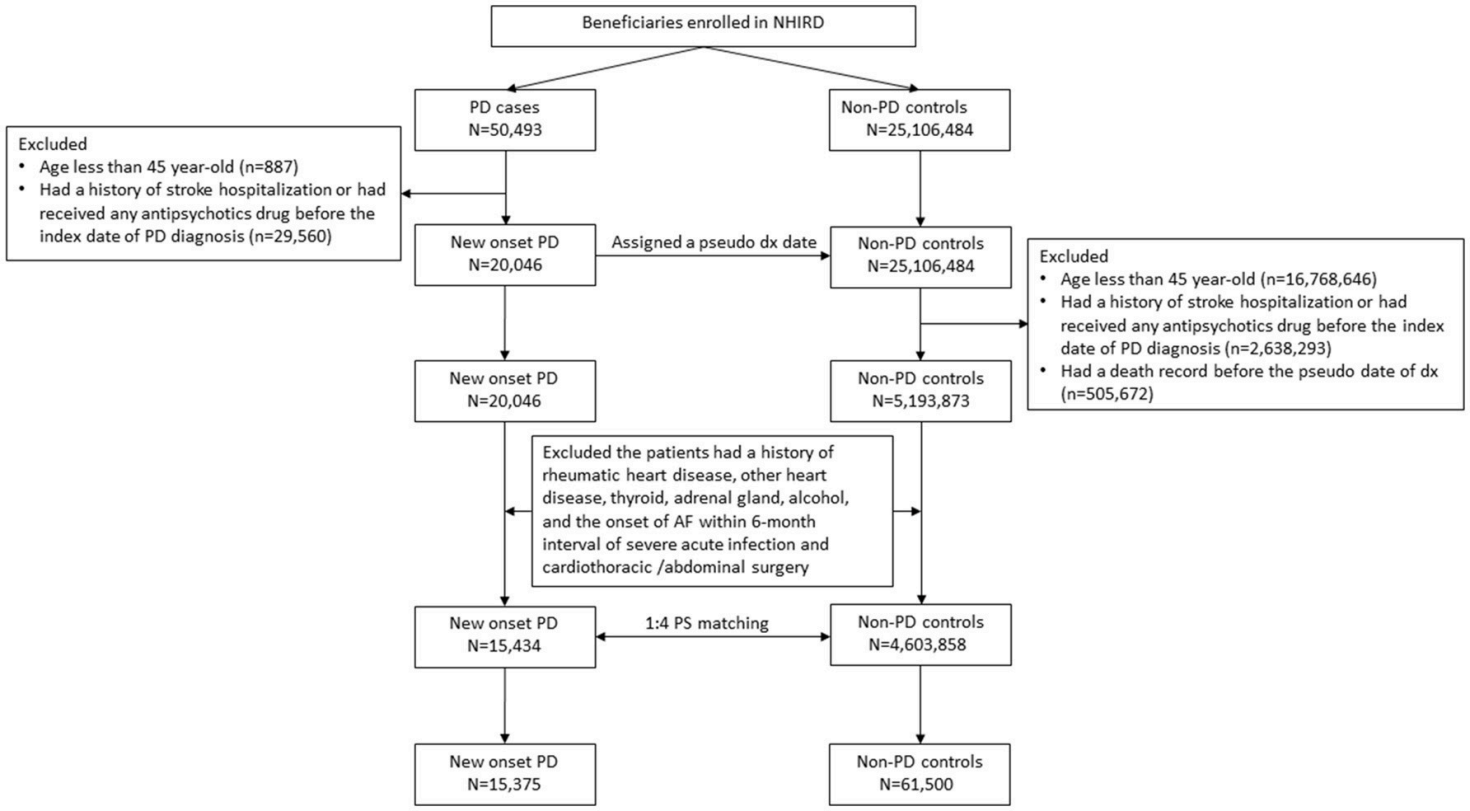
Flowchart of patient selection flowchart. PD, Parkinson's disease and AF, atrial fibrillation. Propensity score, PS.

Both the people with PD and controls were tracked or followed up for their risk of AF according to the study design. Patients with AF were defined as those who first had at least two diagnostic claims (ICD-9-CM: 427.31) and prescription claims for warfarin or oral anticoagulant agents without claims for venous thrombosis (ICD-9-CM: 453), pulmonary embolism (ICD-9-CM: 415, valvular replacement (ICD-9 procedure codes of 35), and antiphospholipid syndrome (ICD-9-CM: 286.53 and 286.59). AF, especially paroxysmal AF, is usually under-diagnosed with a certain latent period before diagnosis. Hence, in the case-control study, AF risk was measured within 2 years of PD or before the index date of PD/pseudo-PD diagnosis. In the cohort study, AF risk was measured 2 years after the index date of PD/pseudo-PD diagnosis. The selection process is presented in Figure 1.

### Statistical Analysis

Baseline characteristics were analyzed using standardized mean difference (SMD). SMD > 0.1 indicated non-negligible differences between the two groups. The case-control study for the odds ratio (OR) of AF before PD and within the 2-year interval of PD diagnosis was evaluated using conditional logistic regression, and a cohort study for the subdistribution hazard ratio (SHR) of new-onset AF after PD diagnosis was evaluated using competing risk analysis. Because the participants were at a high risk of mortality, we applied competing risk model analyses to estimate the absolute relative AF risks. The follow-up period for each patient ranged from the index date of PD/pseudo-PD diagnosis to the date of AF diagnosis or death or the end of the observation period (December 31, 2015). All analyses were performed using SAS/STAT version 9.4 (SAS Institute Inc., Cary, NC, USA) and STATA 14 (Stata Corp LP, College Station, TX, USA). A *P* < 0.05 was considered significant.

## Results

Initially, the study included 15,434 newly diagnosed people with PD, among whom 59.2% were men. After the 1 to 4 PSM, 15,375 subjects remained in the study. After PSM, their mean age was 71.7 ± 9.9 years. The prevalence of previous or current comorbidity was 47.8, 20.7, and 2.5% for HTN, DM, and CHF, respectively (Table 1).

**Table 1:** Basic characteristics of Parkinson's disease (PD) or Non-PD before and after Propensity Score Model (PSM).

	**Before PSM**					**After PSM**				
	**PD**		**Non-PD**			**PD**		**Non-PD**		
	***N***	**(%)**	***N***	**(%)**	**SMD**	***N***	**(%)**	***N***	**(%)**	**SMD**
Sample Size	15,434		4,603,858			15,375		61,500		
Male	9,144	(59.2)	2,553,140	(55.5)	0.077	9,113	(59.3)	36,452	(59.3)	<0.001
Age (y), Mean [SD]	71.7	[9.9]	57.4	[10.4]	1.412	71.7	[9.9]	71.7	[9.9]	<0.001
Age group										
45–64	3,444	(22.3)	3,545,590	(77.0)	1.307	3,443	(22.4)	13,773	(22.4)	<0.001
65	11,990	(77.7)	1,058,268	(23.0)	1.307	11,932	(77.6)	47,727	(77.6)	<0.001
Diagnostic year										
2004–2005	3,812	(24.7)	1,124,015	(24.4)	0.006	3,795	(24.7)	15,180	(24.7)	<0.001
2006–2007	3,955	(25.6)	1,178,943	(25.6)	0.002	3,943	(25.7)	15,772	(25.7)	<0.001
2008–2009	3,869	(25.0)	1,162,550	(25.2)	0.004	3,854	(25.0)	15,416	(25.0)	<0.001
2010–2011	3,798	(24.6)	1,138,350	(24.7)	0.001	3,783	(24.6)	15,112	(24.6)	<0.001
Comorbidity, yes										
HTN	7,398	(47.9)	927,199	(20.1)	0.614	7,355	(47.8)	29,713	(48.3)	0.010
DM	3,206	(20.8)	430,198	(9.3)	0.324	3,175	(20.7)	12,586	(20.5)	0.005
Hyperlipidemia	2,602	(16.9)	429,938	(9.3)	0.224	2,582	(16.8)	10,014	(16.3)	0.014
CHF	460	(3.0)	28,559	(0.6)	0.178	426	(2.8)	1,524	(2.5)	0.018
CAD	2,429	(15.7)	210,937	(4.6)	0.376	2,393	(15.6)	9,579	(15.6)	<0.001
CLD	2,316	(15.0)	241,166	(5.2)	0.328	2,283	(14.8)	9,089	(14.8)	0.002
Renal disease	660	(4.3)	58,419	(1.3)	0.184	644	(4.2)	2,362	(3.8)	0.018
Inflammatory dx	223	(1.4)	34,141	(0.7)	0.068	215	(1.4)	651	(1.1)	0.031
Statin prescription before index date	1,329	(8.6)	186,817	(4.1)	0.188	1,314	(8.5)	5,089	(8.3)	0.010

*SMD (standardized mean difference) indicated the variable difference in means or proportions divided by standard error; imbalance defined as absolute value >0.1 dx, diagnosis; PD, Parkinson disease; HTN, hypertension; DM, diabetes mellitus; CHF, congestive heart failure; CAD, coronary heart disease; CLD, chronic lung disease*.

**Figure 2:**
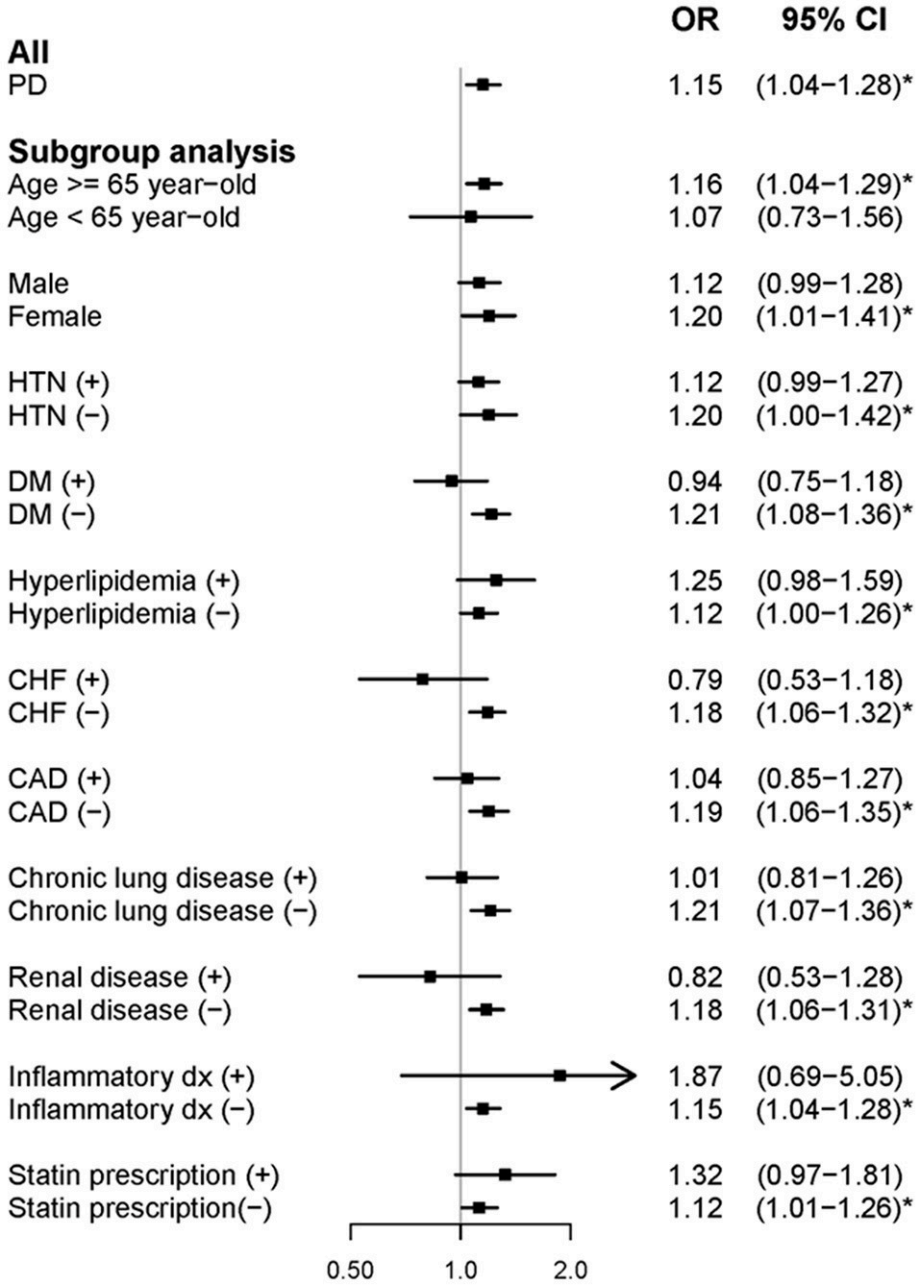
Forest plot showing the adjusted odds ratio of atrial fibrillation among the study participants and subgroup analysis in the case-control study. aOR, adjusted odds ratio; PD, Parkinson's disease; HTN, hypertension; DM, diabetes mellitus; CHF, congestive heart failure; and CAD, coronary heart disease.

In the case-control study, compared with matched controls, newly diagnosed people with PD were significantly comorbid with AF (adjusted OR [aOR]: 1.15, 95% confidence interval [CI]: 1.04–1.28) (Figure 2). According to the subgroup analysis, elderly people (age ≥ 65 years) and women with PD were more likely to have AF. The remainder of the subgroup analysis focused on the effect of each confounding factor of AF on the association between PD and AF. People with PD were only comorbid with AF significantly in the absence of the following factors: HTN (aOR: 1.20, 95% CI: 1.00–1.42); DM (aOR: 1.21, 95% CI: 1.08–1.36); hyperlipidemia (aOR: 1.12, 95% CI: 1.00–1.26); CHF (aOR: 1.18, 95% CI: 1.06–1.36); CAD (aOR: 1.19, 95% CI: 1.06–1.35); chronic lung disease (aOR: 1.21, 95% CI: 1.07–1.36); renal disease (aOR: 1.18, 95% CI: 1.06–1.31); inflammatory disease (aOR: 1.15, 95% CI: 1.04–1.28); and statin prescription (aOR: 1.12, 95% CI: 1.01–1.26). These results indicated that the association between PD and AF was more notable when the confounding factors of AF were excluded.

**Figure 3:**
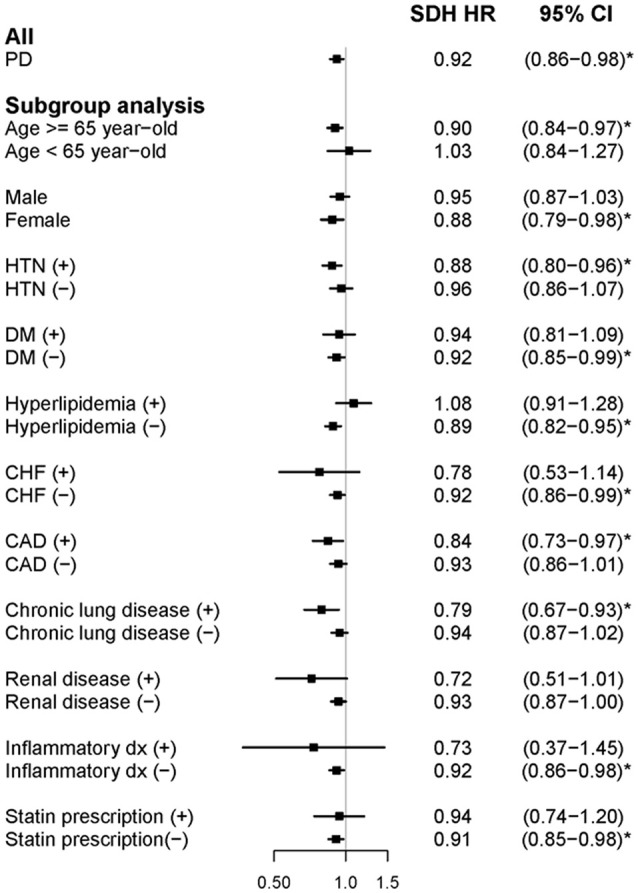
Forest plot showing the adjusted subdistribution hazard ratio of atrial fibrillation among the participants and subgroup analysis in the cohort study. aSDH HR, adjusted subdistribution hazard ratio; PD, Parkinson's disease; HTN, hypertension; DM, diabetes mellitus; CHF, congestive heart failure; and CAD, coronary heart disease.

In the cohort study, newly diagnosed people with PD had lower AF risk during the follow-up period (adjusted SHR: 0.92, 95% CI: 0.86–0.98) (Figure 3). In the subgroup analysis, elderly people and women with PD exhibited a similarly significant risk reduction. However, unlike in the case-control study, the remainder of the subgroup analysis did not reveal a consistent association between AF and the presence of confounding factors of AF. People with PD who had comorbid HTN, CAD, or chronic lung disease were at a greater risk of developing AF. Furthermore, people with PD without DM, CHF, renal disease, inflammatory disease, or statin prescription were more likely to develop AF.

## Discussion

This study demonstrated that people with PD were more likely to be comorbid with AF before and during the onset of motor symptoms. By contrast, people with PD were at a lower risk of AF in the later stages of PD. This discrepancy in the temporal relationship between the two diseases indicates that, similar to other ANS symptoms, AF may be an early NMS of PD.

ANS dysfunction is a common feature in the premotor and early stages of PD (11). For example, constipation occurs before the onset of motor symptoms of PD (4), and it is attributed to ANS degeneration. In addition, heart rate variability (HRV), another parameter of ANS function, is also affected before the onset of motor symptoms of PD. HRV has been found to decrease in people with RBD (12), and decreased HRV was associated with an increased PD risk in a community-based cohort (13). The association between ANS dysfunction and PD risk is supported by pathological findings. A postmortem study of people without Parkinsonism features found that 17% had α-synuclein pathology in the CNS. Among these people, the majority had alterations in the ANS, such as sacral parasympathetic nuclei, myenteric plexus of esophagus, sympathetic ganglia, and the vagus nerve (14). Anatomically, the vagus nerve is smaller in people with PD as measured through ultrasonography, which indicates potential degeneration and neuronal loss (15).

ANS plays an essential role in the genesis and maintenance of AF, and both the sympathetic and parasympathetic nerves regulate cardiac rhythm through the paravertebral sympathetic ganglia and vagal nerve, respectively (16). Unlike cardiac structural lesion–related AF, most idiopathic or lone AF (people with AF but without any other risk factor) is related to ANS dysfunction. For example, abnormal vagal tone, either elevated or suppressed, contributes to vagal AF. The Euro Heart Survey found that vagal AF accounted for 6% of people with paroxysmal AF (17). Vagal AF usually happens after eating or at night and without the adrenergic triggers. Moreover, athletes, especially endurance runners, were five times more likely to develop vagal AF in one cohort of a cross-country study (18). Because of the role of ANS in triggering AF, ganglionated plexi ablation can be used in the treatment of paroxysmal and persistent AF though autonomic denervation (19).

Using a nationwide population-based method, this study demonstrated the association between AF and newly diagnosed PD, finding that newly diagnosed people with PD were more likely to be comorbid with AF before and during the onset of motor symptoms. This association and temporal relationship support the hypothesis of caudal–rostral spreading of α-synuclein pathology, which indicates that the medullary vagal nucleus is the first region in the CNS to be involved in such pathology, followed by the onset of motor symptoms parallel to α-synuclein accumulation in the midbrain. Conversely, they were at lower risk of AF during the follow-up period. After the diagnosis of PD, people tend to have a healthier lifestyle and regular medical check on the blood pressure, blood glucose, and lipid profile, which resulted in better general health condition than population and contribute to the reduction of the risk of AF. It was a limitation of NHIRD study that we could only match by the presence of disease claim without awareness of the severity of the disease. Meanwhile, in most of the occasion, AF is only detected when it became symptomatic, such as tachycardia or leading to stroke. People with PD, especially those with prominent tremor, may prescribe beta-blockers for controlling the tremor, which may mask the AF-induced tachycardia and under-diagnosed the AF.

A notable contribution of this study is identification of the temporal association between AF and PD. Although the prevalence of AF and PD is low, they remain major public health concerns. Moreover, as an ANS-related NMS of premotor PD, AF can serve as one of the predictive biomarkers of PD in the same manner as RBD, anosmia, and constipation, thereby improving the prediction accuracy of PD onset. For further future study to identify the association between AF with PD in the pre-motor stage, it is suggested that for people at high risk of PD (such as RBD or anosmia), the wearable applications should be considered to identify the AF or other kinds of cardiac arrhythmia in a longer-term, more comprehensive manner.

However, this study has several limitations. AF in some patients may be paroxysmal and asymptomatic, which results in delayed diagnosis. Thus, the temporal relationship between AF and PD may be biased. People with early motor symptoms of PD may visit clinics more frequently and receive more examinations, which increases the possibility of asymptomatic AF diagnosis. To eliminate this bias, this study adjusted the potential effect of the frequency of clinic visits when examining the association between AF and PD. In addition, many confounding factors occur in AF genesis, such as conventional vascular risk factors, structural heart disease, and systemic illnesses. This study did not investigate lone AF, which is the most ANS-related AF, because patients with lone AF are rare even in the NHRID database. Nevertheless, a subgroup analysis was performed that found that the association between PD and AF was significant in the absence of any one of them. Therefore, minimizing the confounding factors of AF may strengthen association between the two diseases. Another limitation is that although PD diagnosis has been validated with satisfactory accuracy, there is a possibility of false classification of AF and PD in claims-based medical database research (20). Moreover, some lifestyle factors, such as smoking and coffee drinking or diseases without specific coding (e.g., RBD), which considerably affect PD risk, cannot be analyzed using a claims-based medical database (21). Although AF increases ischemic stroke risk, this study excluded patients with stroke history before PD diagnosis, which avoided possible bias from vascular parkinsonism. In our cohort, the mean age of PD diagnosis was 71.7 years, which was older than the typical onset age. However, a recent meta-analysis of the 17 epidemiological studies showed that the mean age of PD diagnosis was 71.6 years and the peak range of age was 70–79 years, which was similar to our cohort (22).

In conclusion, this study revealed that AF may be an NMS of PD, and people with PD were comorbid with AF before and during the onset of motor symptoms. Future studies may include AF as a premotor biomarker to develop a comprehensive PD prediction model.

## Author Contributions

Research project: **(A)** Conception: all. Organization: C-TH, LC, and L-NC. Execution: C-TH, LC, DW, and L-NC.

Statistical Analysis: **(A)** Design: C-TH, DW, and W-TC. **(B)** Execution: W-TC and L-NC. **(C)** Review and Critique: all.

Manuscript: **(A)** Writing of the first draft: C-TH, LC, and DW. **(B)** Review and Critique: all.

### Conflict of Interest Statement

The authors declare that the research was conducted in the absence of any commercial or financial relationships that could be construed as a potential conflict of interest.
