# Supplementary material for: Association Between Parkinson's Disease and Atrial Fibrillation: A Population-Based Study
Source: Front Neurol. 2019 Feb 5;10:22. doi: 10.3389/fneur.2019.00022 (PMC6370731; doi:10.3389/fneur.2019.00022)
Supplement: Supplementary file 1 [file Table_1.DOCX]

Supplementary Table

Table 1. The logistic regression to estimate the effect of each factor on AF in propensity score model (PSM)

| Factors in the PSM | Coefficient | Odds ratio | (95% Confidence interval) | P-value |
| --- | --- | --- | --- | --- |
| Hypertension | 0.99 | 2.69 | (2.59-2.79) | <.0001 |
| Diabetes | 0.39 | 1.47 | (1.41-1.54) | <.0001 |
| Hyperlipidemia | -0.04 | 0.96 | (0.91-1.01) | 0.114 |
| Congestive heart failure | 0.59 | 1.80 | (1.63-1.98) | <.0001 |
| Coronary heart disease | 0.67 | 1.95 | (1.86-2.04) | <.0001 |
| Chronic lung diseases | 0.71 | 2.03 | (1.93-2.14) | <.0001 |
| Renal disease | -0.03 | 0.97 | (0.89-1.07) | 0.552 |
| Inflammatory dx | 0.44 | 1.56 | (1.37-1.78) | <.0001 |
| Statin prescription | -0.02 | 0.98 | (0.92-1.05) | 0.535 |
